# 1717. *In Vitro* Activity of Aztreonam-Avibactam Against Enterobacterales Isolated from Pediatric and Adult Patients Collected During the ATLAS Global Surveillance Program, 2017-2020

**DOI:** 10.1093/ofid/ofac492.1347

**Published:** 2022-12-15

**Authors:** Mark Estabrook, Francis Arhin, Daniel F Sahm

**Affiliations:** IHMA, Schaumburg, Illinois; Pfizer, Inc., Kirkland, Manitoba, Canada; IHMA, Schaumburg, Illinois

## Abstract

**Background:**

The rapid spread of antimicrobial resistance among clinically isolated Enterobacterales (Eba) continues to threaten public health. Aztreonam (ATM) is a monobactam stable to hydrolysis by metallo-β-lactamases (MBLs) and avibactam (AVI) inhibits class A, class C, and some class D serine β-lactamases. ATM-AVI is being developed for use against drug-resistant isolates of Eba, especially those co-producing MBLs and other β-lactamases. This study evaluated the *in vitro* activity of ATM-AVI and comparators against Eba collected in 2017-2020 from pediatric and adult patients as part of the ATLAS global surveillance program.

**Methods:**

Non-duplicate clinical Eba isolates were collected from 239 sites in 55 countries in Europe, Latin America, Asia/Pacific (excluding mainland China and India), and Middle East/Africa. Susceptibility testing was performed by CLSI broth microdilution and interpreted using CLSI 2022 breakpoints. PCR and sequencing were used to determine the β-lactamase genes present in all isolates with meropenem MIC >1 µg/mL, and *Escherichia coli*, *Klebsiella* spp. and *Proteus mirabilis* with ATM or ceftazidime MIC >1 µg/mL.

**Results:**

MIC_90_ values for ATM-AVI of 0.12 µg/ml were observed for Eba isolates collected from both pediatric and adult patients. Against all Eba isolates, ≤8 µg/ml of ATM-AVI was sufficient to inhibit 99.97% (pediatric) and 99.95% (adult), whereas ATM alone inhibited only 72.0% and 75.9% of these isolates at ≤8 µg/ml, respectively (table). Among isolates that screened positive for an MBL, MIC_90_ values were 0.25 µg/ml (pediatric) and 0.5 µg/ml (adult). Among MBL-positive isolates, ATM-AVI inhibited 100% (pediatric) and 99.9% (adult) at concentrations ≤8 µg/ml. In contrast, ATM alone only inhibited 19.0% (pediatric) and 25.3% (adult) of isolates carrying MBLs at ≤8 µg/ml.

**Figure:**
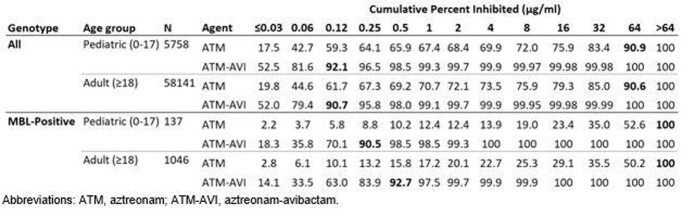

**Conclusion:**

Based on MIC_90_ values, ATM-AVI demonstrated potent *in vitro* activity against Eba isolated both from pediatric and adult patients. The capability of AVI to potentiate ATM against MBL-positive isolates warrants its continued development.

**Disclosures:**

**All Authors**: No reported disclosures.

